# Fe_3_O_4_-PAA–(HP-γ-CDs) Biocompatible Ferrimagnetic Nanoparticles for Increasing the Efficacy in Superparamagnetic Hyperthermia

**DOI:** 10.3390/nano12152577

**Published:** 2022-07-27

**Authors:** Costica Caizer, Isabela Simona Caizer, Roxana Racoviceanu, Claudia Geanina Watz, Marius Mioc, Cristina Adriana Dehelean, Tiberiu Bratu, Codruța Soica

**Affiliations:** 1 Department of Physics, Faculty of Physics, West University of Timisoara, 300223 Timisoara, Romania; costica.caizer@e-uvt.ro (C.C.); isabela.caizer@umft.ro (I.S.C.); 2 Department of Plastic and Reconstructive Surgery, Faculty of Medicine, “Victor Babes” University of Medicine and Pharmacy of Timisoara, 300041 Timisoara, Romania; tiberiu.bratu@umft.ro; 3 Department of Clinical Practical Skills, Faculty of Medicine, “Victor Babes” University of Medicine and Pharmacy of Timisoara, 300041 Timisoara, Romania; 4 Department of Pharmaceutical Chemistry, Faculty of Pharmacy, “Victor Babes” University of Medicine and Pharmacy of Timisoara, 300041 Timisoara, Romania; marius.mioc@umft.ro (M.M.); cadehelean@umft.ro (C.A.D.); codrutasoica@umft.ro (C.S.); 5 Research Centre for Pharmaco-Toxicological Evaluation, “Victor Babes” University of Medicine and Pharmacy of Timisoara, 300041 Timisoara, Romania; 6 Department of Pharmaceutical Physics, Faculty of Pharmacy, “Victor Babes” University of Medicine and Pharmacy of Timisoara, 300041 Timisoara, Romania

**Keywords:** Fe_3_O_4_-PAA nanoparticles, HP-γ-cyclodextrins, nanobioconjugates, superparamagnetic hyperthermia, human HaCaT cells, cell viability, MTT assay

## Abstract

In this paper, we present the obtaining of Fe_3_O_4_-PAA–(HP-γ-CDs) ferrimagnetic nanobioconjugates (PAA: polyacrylic acid, HP-γ-CDs: hydroxypropyl gamma-cyclodextrins) in a hybrid core-shell biostructure (core: inorganic Fe_3_O_4_ nanoparticles, and shell: organic PAA–(HP-γ-CDs)) and their use in superparamagnetic hyperthermia without cellular toxicity and with increased efficacy for future alternative cancer therapy. In order to design the optimal experimental conditions for obtaining nanobioconjugates and then superparamagnetic hyperthermia (SPMHT), we used molecular docking simulation and computational assessment of the maximum specific loss power (SLP) that led to nanoparticles’ heating. The nanoparticles and nanobioconjugates obtained were studied and characterized by X-ray diffraction (XRD), transmission electron microscopy (TEM), Fourier transformed-infrared spectroscopy (FT-IR), dynamic light scattering (DLS), and magnetic measurements (MMs). The cell viability of the nanoparticles and nanobioconjugates was assessed by means of the MTT assay using human immortalized keratinocytes (HaCaT) as an in vitro model. Superparamagnetic hyperthermia with nanoparticles and nanobioconjugates was obtained experimentally in a magnetic field of 15.92 kA/m and frequency of 312.2 kHz for the magnetic nanoparticle core with a (average) diameter of 15.8 nm, which resulted in the maximum hyperthermic effect that led to a temperature of ~42.5 °C necessary in the therapy of tumors in a short time so as not to affect healthy tissues. The biological screening of Fe_3_O_4_-PAA nanoparticles and PAA–(HP-γ-CDs) nanobioconjugates showed no cytotoxic effect on HaCaT cells for a time interval of 24 h, both under standard (37 °C) and hyperthermia conditions (42.5 °C). Thus, Fe_3_O_4_-PA–(HP-γ-CDs) ferrimagnetic nanobioconjugates can be used successfully in superparamagnetic hyperthermia without toxicity and with increased efficiency due to the small layer thickness of the PAA–(HP-γ-CDs) shell, which is suitable in this alternative therapeutic technique.

## 1. Introduction

The hyperthermic approach of tumorigenic cells is considered a hot topic, with technology under development [1]. In this regard, extensive research has been conducted in the magnetic-mediated hyperthermia field due to several advantages when implemented in vivo, such as a minimally invasive procedure and tumor-localized cytotoxicity, by taking advantage of the higher susceptibility of cancer cells to elicit cellular damages under hyperthermic conditions compared to healthy cells that are exposed to the same hyperthermic treatment [2]. This aspect could be related to the higher metabolic rate developed by cancer cells [3], but it could also be caused by the more acidic microenvironment and inefficient oxygen concentration encountered within cancer cells [4].

The mechanism involved in the cancer cells’ destruction through magnetically induced hyperthermia is based on magnetic relaxation mechanisms: Néel relaxation and/or Brownian relaxation. However, regarding the cellular mechanism leading to hyperthermia cytotoxicity, lysosome-mediated pathways are considered to play a key role. Furthermore, in addition to the thermal conditions known to induce cellular damage, the amplitude of the alternating magnetic field induces important cellular alterations [5].

However, even if, theoretically, this approach assures promising applicability for alternative cancer therapy, several aspects should be respected when developing magnetic nanoparticles as vectors for biocompatible hyperthermia:

(a) The type, size, and the surface of the magnetic particles: in this regard, nanoparticles ranging in size between 10 and 100 nm are considered suitable for in vivo administration [3]. Additionally, these nanoparticles may use the enhanced permeability and retention (EPR) effect developed by several types of tumors [6,7], a phenomenon that plays a key role in the clinical outcome [8]. In addition, when administrated at the tumor site, magnetic nanoparticles could provide insightful data on the EPR effect of tumor tissue because, as demonstrated by Chen et al. [8], the higher the contrast of the tumor tissue after the injection of magnetic nanoparticles (used as contrast agents), the better the EPR effect is represented at the tumor site. Knowing this aspect, it may lead to a better understanding of magnetic nanoparticles’ mechanism of action within the tumor tissue. On the other hand, particles with a diameter above 200 nm are prone to phagocytosis while particles with a diameter less than 10 nm are removed by the renal system, which are key factors when selecting the nanoparticle size for biomedical applications [3,9]. Moreover, in order to avoid a cytotoxic effect related to the aggregation phenomenon, metallic nanoparticles should possess a zeta potential close to −30 mV [1]. Additionally, the EPR effect is facilitated by highly negatively charged particles [10];
(b) The heat dissipation features of the magnetic nanoparticles (MNPs): this aspect is of major significance for magnetically induced hyperthermia, as the newly developed MNPs should be able to induce hyperthermic conditions (around 43 °C) in the presence of an alternating magnetic field when applied under nontoxic parameters (magnetic field frequency between 100 and 300 kHz) [3]. Moreover, the superparamagnetic characteristics of MNPs offer superior advantages for biomedical applications, as according to Langevin’s theory related to paramagnetism, superparamagnetic NPs elicit high saturation magnetization when an external magnetic field is applied while no remanent magnetization is developed by MNPs when the magnetic field is removed [3,4,11]. This feature may be of real interest when administrated in vivo, as magnetic dipolar interaction may be avoided, and thus thrombus-related events are minimal [3].

Moreover, in magnetic hyperthermia (with large soft magnetic nanoparticles (>20–25 nm)) or superparamagnetic (with small soft magnetic nanoparticles (<20–25 nm)) for alternative cancer therapy, the most important issues today remain the identification of (i) the most suitable magnetic nanoparticle (type, size, shape) and (ii) the organic layer that coats the nanoparticles on their surface that to leads to increased efficiency and reduction or even elimination of their cellular toxicity in this therapy. Among the various types of MNPs, iron oxide nanoparticles (NPs), such as Fe_3_O_4_ NPs (magnetite), are considered one of the most promising candidates for biomedical applications such as magnetic-mediated hyperthermia due to the superparamagnetic behavior [12] and especially due to their biocompatibility and biodegradability at small sizes [13]. Nevertheless, Fe_3_O_4_ NPs are usually coated with different hydrophilic layers to ensure a lack of interactions between the nanoparticles (van der Waals and magnetic dipole-dipole, which lead to agglomerates) and the colloidal stability [7]. Fe_3_O_4_ ferrimagnetic nanoparticles smaller than 30–50 nm have been found to be targeted for magnetic hyperthermia and superparamagnetic hyperthermia [14,15,16,17,18,19,20,21,22,23], but many other studies have been carried out to find other suitable ferrimagnetic nanoparticles or those more suitable than magnetite, both in terms of thermal efficiency and cell toxicity [24,25,26,27,28,29,30]. Research in this field has also focused on ferromagnetic nanoparticles [31,32], with special attention given to Fe nanoparticles, which in terms of efficiency of loss power and heating would be most suitable due to their high magnetization and initial susceptibility [33] compared to ferrimagnetic nanoparticles [34]. However, these nanoparticles involve higher toxicity problems than ferrimagnetic ones; however, once this issue is solved by their very good biocompatibility with the biological environment, using modern nanobiotechnology, it would be possible to use them in magnetic hyperthermia with higher efficiency than the ferrimagnetic nanoparticles used so far in this field.

In magnetic hyperthermia, the biocompatibility of magnetic nanoparticles is necessary to avoid toxicity to healthy cells and their safe use in vivo, both when injected to reach the tumor and during magnetic hyperthermia, and after therapy [35]. Additionally, from the point of view of the efficiency of magnetic hyperthermia, the organic shell that coasts the magnetic nanoparticles via different techniques (surfactating, coating, bioencapsulation, bioconjugation, biofunctionalization, etc.) using different organic chemical agents [22,36] is necessary to avoid the formation of large bionanostructures, which would lead to inefficiency in magnetic hyperthermia for obtaining the temperatures of 42–43 °C required in tumor therapy. In addition, the warm-up time should be as short as possible to avoid damaging healthy tissues. Moreover, magnetic nanoparticles coated with these organic structures must be able to disperse very well in water (or in a pharmaceutical liquid) in order to form very good and stable suspensions for a long time.

Regarding the biocompatibility issue of magnetic nanoparticles and the lowest possible thickness of the organic layer on the surface of nanoparticles, we propose the use cyclodextrins [37] to cover magnetic nanoparticles as a new strategy in the field of magnetic hyperthermia that leads to better results in this field [38]. Therefore, we chose gamma-cyclodextrins (γ-CDs), which are cyclic oligosaccharides with 8 glucose units in a ring, forming a cone-shaped torus with a cavity depth of only ~0.8 nm [37]. γ-CDs are currently used in the pharmaceutical, drug delivery, and food industry [37,39,40,41], having very good stability over time [38]. The small size of the γ-CDs allows a high increase in the volume packing fraction of magnetic nanoparticles in the sample for magnetic hyperthermia, leading to increased thermal efficiency (heating to the required temperature in magnetic hyperthermia in a short time). At the same time, the van der Waals and magnetic dipole–dipole interactions, which lead to the formation of large agglomerates of magnetic nanoparticles, are eliminated [42,43], with a beneficial effect on magnetic hyperthermia of increasing the efficiency and reducing toxicity.

Thus, in this paper, we present an experimental study based on our theoretical predictions [23,44] regarding superparamagnetic hyperthermia and warming temperatures, using ferrimagnetic nanoparticles of Fe_3_O_4_ bioconjugated with hydroxy-propyl gamma-cyclodextrins (HP-γ-CDs) through poly(acrylic acid) (PAA) adsorbed on the surface of nanoparticles, prepared by us. For the bioconjugation of magnetic nanoparticles with γ-CDs, we found the use HP-γ-CDs was more appropriate due to an increased affinity of PAA for branched HP-γ-CDs. Additionally, we considered the identification of the most suitable Fe_3_O_4_ nanoparticles in terms of their size and shape for use in superparamagnetic hyperthermia, parameters that strongly influence the hyperthermic effect reflected in the specific loss power (SLP) and heating temperature (ΔT) presented in the paper. Considering these factors, superparamagnetic hyperthermia using the nanobioconjugates of Fe_3_O_4_-PAA–(HP-γ-CDs) obtained by us was tested in vitro on a human immortalized keratinocytes cell line (HaCaT) in order to obtain the hyperthermic effect (temperature of 42.5 °C) necessary in tumor therapy, and cellular viability assessment using the MTT colorimetric test was carried out.

In summary, the aim of the current study was: (i) the synthesis of Fe_3_O_4_-PAA ferrimagnetic nanoparticles, and Fe_3_O_4_-PAA–(HP-γ-CDs) ferrimagnetic nanobioconjugates with very good heat dissipation features; (ii) evaluation of the physicochemical characteristics of the Fe_3_O_4_-PAA and Fe_3_O_4_-PAA–(HP-γ-CDs) samples to confirm the heat dissipation characteristics and the formation of a stable magnetic suspension; and (iii) screening of the biological profile of the magnetic samples both under standard and magnetically induced hyperthermic conditions by employing an in vitro model based on healthy human HaCaT cells using one of the most consecrated in vitro toxicological assessment: the MTT assay.

## 2. Materials and Methods

### 2.1. Molecular Docking Analysis

Molecular docking analysis was performed using PyRx 0.8 (The Scripps Research Institute, La Jolla, CA, USA). For this purpose, a fragment of polyacrylate containing 20 repetitive monomer units (PA20n) was used as a ligand. The ligand geometry optimization was carried out using Avogadro, with the MMFF94 theoretical force field. The molecular structures of various CDs (β-CD, γ-CD, hydroxypropyl β-CD (HPBCD), hydroxypropyl γ-CD (HPGCD), Heptakis (6-deoxy-6-(2-carboxyetil)-thio)–gamma-cyclodextrin-Mono-OH-SGM) were sketched as 2D mol files with the Biovia Draw software (version 20.1.0.258, Dassault Systemes Biovia, San Diego, CA, USA, 2022). Subsequently, these structures were geometrically optimized under the same conditions used in the case of the ligand. Finally, the CDs were prepared as suitable targets for molecular docking, using Autodock Tools, version 1.5.6. (The Scripps Research Institute, La Jolla, CA, USA, 2022). The P20n ligand was docked into the internal cavity of the CDs using the imbedded Vina module of PyRx [45]. The results were recorded as free binding energy (∆G, kcal/mol). CD–ligand interactions were analyzed using Accelerys Discovery Studio 4.1 (Dassault Systemes Biovia).

### 2.2. Computational Study Method on the Specific Loss Power in Superparamagnetic Hyperthermia

The specific loss power in superparamagnetic hyperthermia was studied by computational simulation using TableCurve 3D professional software. The input parameters were the characteristic sizes of the magnetic nanoparticles used in the study, the thickness of the organic layer on the surface of the nanoparticles bioconjugated with gamma-cyclodextrins, the viscosity of the nanoparticle dispersion medium, and the volumetric packing fraction of the nanoparticles in the sample. The observable output studied was the specific loss power (SLP) as a function of the size (average diameter) of the Fe_3_O_4_ nanoparticles (D) and the frequency (f) and amplitude of the alternating magnetic field (H) used to obtain superparamagnetic hyperthermia. The values and ranges for the characteristic sizes and parameters considered in this study are given in Table 1. The value for the volume packing fraction (ε) and the diameter range of the nanoparticles (D) were established based on the data used in magnetic hyperthermia. The parameters of the alternative magnetic field (H,f) were established according to the data used by us in the experimental section, and the value of the parameter d resulted from our experimental data. The other observables in Table 1 (M_s_, K, ρ, η) are known in the literature [34,46,47].

The specific loss power (*SLP*) was numerically calculated using the following formula [44]:

(1) $$SLP=\frac{3\pi \mu_{0}\chi_{i}}{\rho \xi }\left(\coth\xi -\frac{1}{\xi }\right)\frac{2\pi f\frac{\tau_{N\;}\tau_{B\;}}{\tau_{N\;}+\tau_{B\;}}}{1+{\left(2\pi f\frac{\tau_{N\;}\tau_{B\;}}{\tau_{N\;}+\tau_{B\;}}\right)}^{2}}fH^{2}\;\;\;\;\;\;\left(W/g\right)$$

for W/g units, where *χ_i_* is the initial magnetic susceptibility of magnetic nanoparticles:

(2) $$\chi_{i}=\frac{\varepsilon \pi \mu_{0}M_{s}^{2}D^{3}}{18k_{B}T}$$

where *ξ* is the Langevin parameter [48,49,50]:

(3) $$\xi =\frac{\pi \mu_{0}M_{s}D^{3}}{6k_{B}T}H\;\;$$

where *τ_N_* is the Néel relaxation time [51]:

(4) $$\tau_{N}=\tau_{0}\cdot exp\left(\frac{\pi KD^{3}}{6k_{B}T}\right)$$

where *τ_B_* is the Brown relaxation time [52], which in this case will be:

(5) $$\tau_{B\;}=\frac{3\pi \eta {\left(D+2d\right)}^{3}}{6k_{B}T}$$

Other observables from the above equations are as follows: *μ*_0_ is the magnetic permeability of the vacuum, *k_B_* is the Boltzmann’s constant, *T* is temperature, and *τ*_0_ is a time constant that is 10^−9^ s [53].

If the nanoparticles are fixed in the tumor (immobile nanoparticles), the specific loss power in this case is calculated with the formula:

(6) $$SLP_{N}=\frac{3\pi \mu_{0}\chi_{i}}{\rho \xi }\left(\coth\xi -\frac{1}{\xi }\right)\frac{2\pi f\tau_{N\;}}{1+{\left(2\pi f\tau_{N\;}\right)}^{2}}fH^{2}\;\;\;\;\;\;\left(W/g\right)$$

This formula derives from Equation (1) considering *τ_B_* is very high (*τ_B_* >> *τ_N_*), a situation in which magnetic relaxation will occur only through Néel processes characterized by the relaxation time *τ_N_*.

### 2.3. Synthesis Method and Characterization Techniques of Fe_3_O_4_-PAA Magnetic Nanoparticles and Fe_3_O_4_-PAA–(HP-γCDs) Nanobioconjugates

#### 2.3.1. Materials and Synthesis Method

For the synthesis of Fe_3_O_4_-PAA ferrimagnetic nanoparticles and Fe_3_O_4_-PAA–(HP-γ-CDs) nanobioconjugates, the co-precipitation method and the following reagents were used: FeCl_3_·6H_2_O (Fluka AG, Buchs, Switzerland), FeSO_4_·7H_2_O (Sigma Aldrich, Taufkirchen, Germany), NH_4_OH 25% (SC Silal Trading, București, Romania), polyacrylic acid (PAA) 5100 (Sigma Aldrich, Germany, Mw~5100), 2-hydroxy-propyl-γ-cyclodextrin (HP-γ-CDs) (Sigma Aldrich), and absolute ethanol (Sigma Aldrich). All chemicals used were of analytical grade.

##### Fe_3_O_4_-PAA Nanoparticles Synthesis

The Fe_3_O_4_ nanoparticles were prepared using the co-precipitation method [54,55]. First, the amounts of each reagent corresponding to 0.01 mol Fe(II) and 0.02 mol Fe(III) were calculated in order to obtain 0.01 mol of Fe_3_O_4_. After weighing, the salts were solubilized in 300 mL of distilled water and then poured into a round-bottom flask. The working atmosphere was inert (argon) to avoid oxidation of the magnetite to maghemite or hematite. The solution thus prepared was stirred and gradually heated to 80 °C, and then ammonia (solution of 25% concentration) was added dropwise. Alongside the ammonia addition in the solution, the formation of a black precipitate was observed. Afterwards, the temperature was increased to 90 °C and was maintained for 20 min. Next, to create a coating on the surface of the formed black precipitate, a solution of sodium salt of polyacrylic acid (PAA) was added (concentration 10%). After completing the synthesis, the sample was rinsed three times with a total volume of 1500 mL deionized water to remove the excess ammonia solution. In order to accelerate the non-magnetic components separation, a magnet was placed under the flask so water could easily be decanted. Next, the sample of Fe_3_O_4_-PAA nanoparticles was dried at 70 °C, hand ground, and stored in containers.

##### Synthesis of Fe_3_O_4_-PAA–(HP-γ-CDs) Nanobioconjugates

For the preparation of nanobioconjugates of Fe_3_O_4_-PAA with HP-γ-CDs, noted Fe_3_O_4_-PAA–(HP-γ-CDs), the commercially available 2-hydroxy-propyl-γ-cyclodextrin (HP-γ-CDs) and the previously synthesized Fe_3_O_4_-PAA NPs were used at a molar ratio of 1:1 (Fe_3_O_4_-PAA:2-hydroxy-propyl-γ-cyclodextrins). The experimental procedure consisted in mixing the sample of Fe_3_O_4_-PAA NPs with the appropriate amount of HP-γ-CDs using a pestle mortar. The mixture formed between the two components was treated with a 70% ethanolic solution, then homogenized, and mixed for about 20 min until a paste with high viscosity was formed. Afterwards, the obtained product was dried in the oven at 70 °C until a constant mass was obtained.

#### 2.3.2. X-ray Diffraction Technique

X-ray diffraction was used in order to establish the phase composition of the synthesized nanoparticles. The diffraction spectra were recorded using a Rigaku UltimaIV diffractometer (Rigaku Corporation, Neu-Isenburg, Germany), at room temperature, radiation Cu-Kα, in the range 10°–70° on the 2θ scale. The average crystallite size was calculated with the Debye–Sherrer equation.

#### 2.3.3. Fourier Transformed Infrared Spectroscopy

The sample was characterized by means of IR spectrophotometry. The Fourier transformed infrared (FT-IR) spectra of the sample were recorded with a Shimadzu IR Affinity-1S spectrophotometer (Shimadzu, Duisburg, Germany) in the range of 400–4000 cm^−1^, using the method of potassium bromide pellets, with a resolution of 4 cm^−1^.

#### 2.3.4. Transmission Electron Microscopy

High-resolution transmission microscopy (HR-TEM) was performed using a Hitachi TEM system (HT7700, Japan) with 100.0 kV, 120k Zoom, HR, and 0.2 nm resolution. For visualization and registered HR-TEM images, the nanoparticles were dispersed in deionized water, and ultrasonicated for 30 min before placing a drop of suspension on the grid of the microscope.

#### 2.3.5. Dynamic Light Scattering and Zeta Potential Measurements

In order to determine the hydrodynamic diameter of the sample, a Vasco Particle Size Analyzer from Cordouan Technologies (Pessac, France) was used. The parameters of the determination were laser power 100%, temperature 25 °C, number of channels 350, time interval 14 µs, DTC position down, and acquisition mode continuous. The measurements were performed in triplicate. The obtained data were plotted and analyzed with the Gauss non-linear curve fit using the OriginPro 2020b software (OriginLab Corporation, Northampton, MA, USA).

For the zeta potential measurement, a Wallis Zeta-potential Analyzer from Cordouan Technologies (Pesac, France) was used. The parameters of the measurement were medium resolution, temperature 25 °C, plastic cuvette, laser power 65%, electrode distance 5 mm, and Henry function Huckel. Ten measurements were performed for each sample.

For the DLS determination and the zeta potential measurements, a suspension was made by sonication (ultrasonic bath) using the synthesized magnetic powder/nanobioconjugates and distilled water.

#### 2.3.6. Magnetic Characterization of Fe_3_O_4_-PAA Nanoparticles for Superparamagnetic Hyperthermia

From the point of view of magnetic hyperthermia, it is very important to know how to magnetize nanoparticles in the magnetic field. To study the magnetic behavior in the external magnetic field of the obtained nanoparticles, and to determine their characteristic magnetic quantities, were used the fluxmeter method and the experimental measurement technique described in [56]. The accuracy in determining the magnetization was 0.82% and that of the magnetic field was 0.24%. The maximum magnetic field used was 1500 Oe.

### 2.4. Materials and Methods for In Vitro Experimental Study

#### 2.4.1. Cell Line and Cell Culture Conditions

In the present study, human immortalized keratinocytes HaCaT cells (300493; CLS Cell Lines Service GmbH, Eppelheim, Germany) were used as an in vitro model to assess the biological profile of different concentrations of the Fe_3_O_4_-PAA nanoparticles and Fe_3_O_4_-PAA-(HP-γ-CDs) nanobioconjugates, under both standard and hyperthermic conditions. The HaCaT cell line was cultured in specific culture medium: DMEM high glucose (4.5 g/L) media, with 15 mM HEPES, and 2 mM L-glutamine (Sigma-Aldrich, Munich, Germany), which was further supplemented with 10% fetal calf serum and 1% antibiotic mixture containing 0.1 mg·mL^−1^ streptomycin: 100 IU·mL^−1^ penicillin to avoid any possible fungal or microbial contamination of the cell cultures. The cell cultures were maintained in a humidified atmosphere enriched with 5% CO_2_ and 37 °C using a Steri-Cycle i160 incubator (Thermo Fisher Scientific, Inc., Waltham, MA, USA). All in vitro techniques were performed under sterile conditions using the MSC Advantage 12 model biosafety cabinet (Thermo Fisher Scientific, Inc., Waltham, MA, USA).

#### 2.4.2. MTT Assay under Standard Conditions

A colorimetric test, the MTT assay, was performed to evaluate the viability percentage of the immortalized human keratinocytes (HaCaT) after exposure to different concentrations (200, 500, and 1000 μg/mL) of test sample (Fe_3_O_4_-PAA and Fe_3_O_4_-PAA-(HP-γ-CDs) by employing 24- and 48-h time exposure intervals. DMEM was used as a vehicle to obtain the stock solutions of the test samples at a concentration of 5 mg/mL Fe_3_O_4_ compound.

Under standard conditions (37 °C), the protocol consisted of seeding 1 × 10^4^ cells/well in 96-well plates. Afterwards, the cells were maintained under standard conditions until the optimal confluence (80%) was reached (approximately 24 h). This step was followed by replacing the old medium with 100 μL/well of fresh medium that contained the test samples at a final concentration of 200, 500, or 1000 μg/mL equivalent in Fe_3_O_4_-PAA NPs. After, the cells were further incubated for a period of 24 and 48 h, respectively.

After the stimulation period ended, the MTT protocol was performed as follows: 10 μL/well MTT reagent was added for a period of 3 h while the culture plates were protected from light. This period of time was used by the viable cells to convert the yellow MTT to dark blue formazan crystals via the mitochondrial reductase pathway. The precipitated formazan crystals were solubilized by the addition of 100 μL/well of lysis buffer solution.

To quantify the cell viability rate, the absorbance of each well was spectrophotometrically measured at the 570-nm wavelength by using a microplate reader (xMark^TM^ Microplate Spectrophotometer, Bio-Rad Laboratories, Hercules, CA, USA).

The negative control was represented by the control cells: the cell population treated with specific growth medium (DMEM). The cell viability (%) of the sample-treated cell culture was normalized to the cell viability (%) of the control cells.

#### 2.4.3. In Vitro Magnetically Induced Hyperthermia (MHT) Protocol

**Figure 1:**
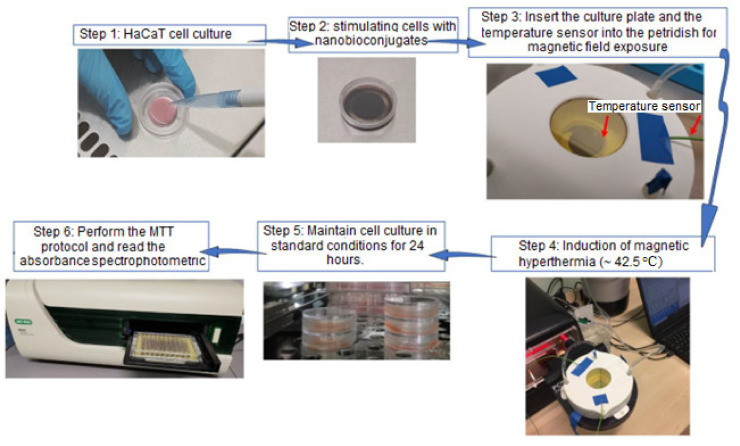
Schematic representation of the working protocol for achieving magnetic hyperthermia in vitro along with the cell viability assessment by means of the MTT test.

The protocol for achieving in vitro magnetic hyperthermia is shown in Figure 1. A density of 5 × 10^5^ HaCaT cells were cultured in a final volume of 800 µL medium and seeded on a sterile 35-mm micro-dish (ibidi, cat no. 80156) until a cell confluence of approximately 80% was obtained (Figure 1-Step 1). Afterwards, the cell monolayer was treated with the sample of interest (Fe_3_O_4_-PAA-(HP-γ-CDs)) (Figure 1-Step 2) and the temperature sensor was inserted inside the culture plate (Figure 1-Step 3). After this step, magnetic hyperthermia was induced by employing the following physical parameters: amplitude of the magnetic field of 15.92 kA/m and frequency of 312.2 kHz, using F3 Driver of 3 kW in power (Figure 1-Step 4). The hyperthermia conditions (42.5 °C) were maintained for approximately 30 min. After this, the culture plate was further incubated under standard conditions (37 °C) until the 24-h interval was reached (Figure 1-Step 5).

In parallel, the same procedure was also employed for HaCaT cultures treated with cell culture medium only.

The cell viability percentage was quantified by employing the MTT protocol (Figure 1-Step 6) as described above; however, the volume of MTT and lysis buffer was adjusted to the micro-dish dimensions. The cell viability percentage was calculated as previously described in one of our studies [57].

### 2.5. Experimental Magnetic Hyperthermia

The experimental achievement of the magnetic hyperthermia, and the recording of the heating temperature of the nanoparticles in suspension and in vitro were carried out using a professional F3 Driver Instrument for Magnetic nanoHeating (D5 series, nanoScale Biomagnetics, Zaragoza, Spain) especially designed for this, with a power of 3 kW. The generator can produce magnetic fields up to 40 kA/m and different frequencies in the range of 100–400 kHz used in magnetic hyperthermia. The driver is also provided with specific inductor coils for calorimetric and in vitro studies, and the conditions required in the experiments can be met. The temperature in the experiments was measured using a temperature sensor with fiber optic. The data acquisition of the driver system allows an accuracy of 0.1 °C when recording the temperature.

## 3. Results and Discussion

### 3.1. Molecular Docking

In order to obtain magnetic nanoparticles–cyclodextrins (CDs) complexes that are compatible for biological use, one should take into account how these nanoparticles can be complexed with CDs. Since there are no large functional groups on the nanoparticle surfaces that can be included in the internal cavity of CDs, it is necessary to functionalize the surface of the magnetic nanoparticles with organic molecules, both to be able to form stable inclusion complexes and to avoid the formation of agglomerations of the synthesized nanoparticles. Following extensive literature research, polyacrylic acid (PAA) emerged as a compound that is capable of satisfying the two aforementioned conditions [54,58]. Furthermore, the ability of polyacrylic acid residues to form stable complexes with different types of CDs was analyzed using a molecular docking approach (Section 2.1). The obtained results are shown in Table 2.

**Figure 2:**
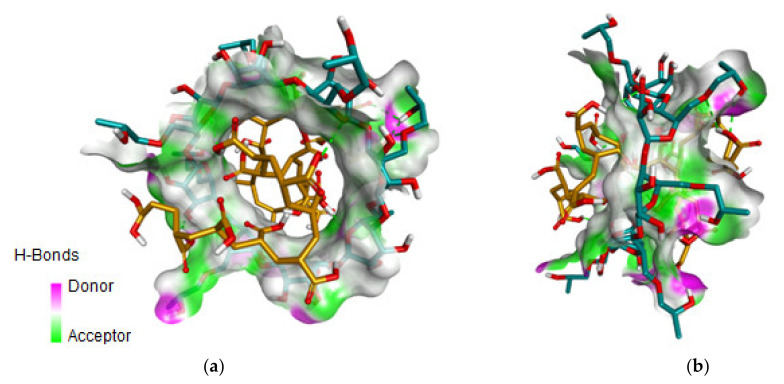
The binding interactions of PAA (orange) formed within the internal cavity of the HP-γ-CDs (turquoise): (**a**) front and (**b**) side representation. The HBs formed are represented as green dotted lines and the internal surface of the cyclodextrin highlights the areas of HB formation.

The obtained results indicate similar affinities of PA20n (PAA) for all target CDs, although an increased affinity for branched CDs is observed. Given the chemical structure of the ligand, PA20n penetrates the internal cavity of the cyclodextrins and forms multiple hydrogen bonds with the CDs’ OH groups, both in the cavity area and at the surface. This binding mode is represented in Figure 2 for the PA20n–HPGCD complex.

Considering the obtained results, the low toxicity of branched cyclodextrins compared to that of non-functionalized CDs, and the commercial availability, we chose HPGCD (noted HP-γ-CDs) as the suitable cyclodextrin to obtain polyacrylate functionalized Fe_3_O_4_-PAA–(HP-γ-CDs) complexes.

### 3.2. Computational Assessment of Specific Loss Power in Superparamagnetic Hyperthermia with Fe_3_O_4_-PAA Nanoparticles

Taking into account the result obtained in the previous section, which shows that the most valid nanobiostructure is Fe_3_O_4_-PAA–(HP-γ-CDs), using 3D computational simulation, we identified the specific loss power (SLP) that leads to the heating of nanoparticles in superparamagnetic hyperthermia using this biostructure, and investigated how SLP depends on the size of the Fe_3_O_4_ nanoparticles (D) and the parameters of the alternating magnetic field: amplitude (H) and frequency (f). Thus, SLP was calculated in two cases (i) when Fe_3_O_4_-PAA–(HP-γ-CDs) nanobioconjugates are dispersed in a pharmaceutical liquid (considered saline), using Equation (1) with Formulas (2)–(5); and (ii) when Fe_3_O_4_-PAA–(HP-γ-CDs) nanobioconjugates are immobile (the case when the nanoparticles are fixed in the tumor). SLP was determined as a function of the diameter of the nanoparticles (D) for the frequency (f) in the range 200–400 kHz and the amplitude of magnetic field (H) of 16 kA/m; these values were previously found by us to be optimal [23]. These values were then provided experimentally by the F3 Driver magnetic hyperthermia generator we used.

**Figure 3:**
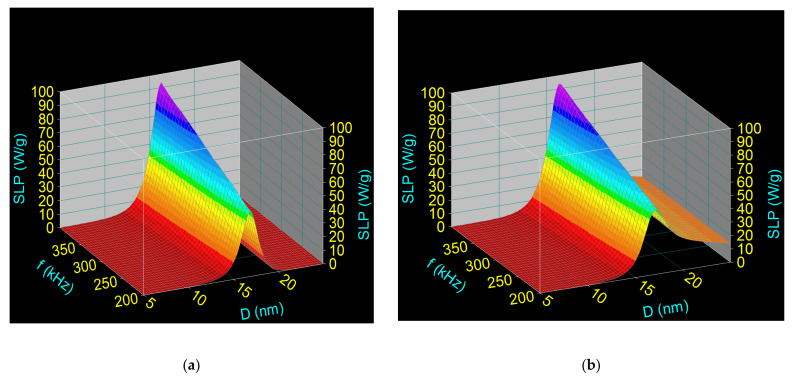
Specific loss power as a function of the nanoparticle diameter and frequency of the magnetic field and H = 16 kA/m, in the cases: (**a**) Fe_3_O_4_-PAA–(HP-γ-CDs) fixed nanoparticles, and (**b**) Fe_3_O_4_-PAA–(HP-γ-CDs) mobile nanoparticles.

Using the values of the parameters in Table 1, the results obtained in the two cases are shown in Figure 3.

The results obtained show a very important aspect in terms of superparamagnetic hyperthermia, namely that regardless of the state of nanoparticles—fixed, mobile, or an intermediate state—the maximum specific loss power (SLP)_M_ obtained is approximately the same in value. This aspect is very important for the practical implementation of superparamagnetic hyperthermia because the maximum power is not affected by the state in which the nanoparticles are found in the tumor: immobile (fixed), slightly mobile, or mobile. Moreover, this result obtained in our case, when the Fe_3_O_4_ nanoparticles were coated with HP-γ-CDs by means of the PAA polymer, is important because it eliminates the still unsolved controversy of researchers’ point of views on the mobility or immobility of nanoparticles in tumors in the case of magnetic hyperthermia, which affects the specific loss power.

**Figure 4:**
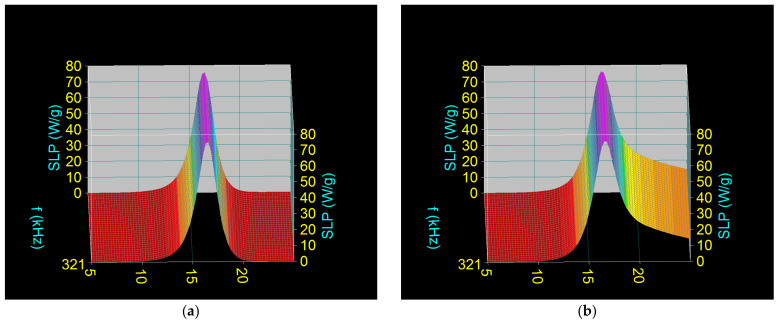
Relaxation contributions to the specific loss power for 321 kHz and 16 kA/m magnetic field: (**a**) Néel relaxation in the case of fixed Fe_3_O_4_-PAA–(HP-γ-CDs) nanobioconjugates, and (**b**) Néel–Brown relaxations in the case of mobile Fe_3_O_4_-PAA–(HP-γ-CDs) nanobioconjugates.

Thus, in our case, regardless of the state of the nanoparticles in the tumor: fixed (Figure 4a) or mobile (Figure 4b), the result regarding the maximum specific loss power (and as a result the heating temperature of the magnetic nanoparticles) does not change. Coating of the nanoparticles with PAA-HP-γ-CDs only affects the shape of the variation of SLP for nanoparticles with a diameter higher than ~16 nm (Figure 4b). However, this has no effect on the maximum power (SLP)_M_, which is of interest in magnetic hyperthermia, as long as the nanoparticle diameter is approximately 16 nm, which is intended to be obtained in practical magnetic hyperthermia.

For different frequencies in the considered range (Figure 3), the optimal diameters of the nanoparticles (Do) that led to the maximum specific loss power (SLP)_M_ in superparamagnetic hyperthermia remain approximately the same in the two cases (Table 3 and Table 4). However, (SLP)_M_ increases significantly with increasing frequency in each of the two cases, with values that led to efficient heating of the nanoparticles in superparamagnetic hyperthermia (obtaining the temperature of 42–43 °C in a short time) [23].

According to the results obtained, in order to obtain the maximum specific loss power in superparamagnetic hyperthermia that leads to efficient heating of Fe_3_O_4_-PAA–(HP-γ-CDs) magnetic nanobioconjugates, it is necessary that the ferrimagnetic core of Fe_3_O_4_ nanoparticles has an optimal diameter of ~16–17 nm, depending on the frequency.

### 3.3. Obtaining Superparamagnetic Fe_3_O_4_-PAA–(HP-γ-CDs) Nanobioconjugates

Based on our theoretical results from Section 3.1 and Section 3.2, the next step in our study was to experimentally obtain nanoparticles with the identified characteristics, which are suitable for use in superparamagnetic hyperthermia with a maximum efficiency and without toxicity. Thus, in the first stage, we obtained Fe_3_O_4_-PAA nanoparticles with the appropriate size, resulting from the analysis in Section 3.2, and in the second stage, we obtained Fe_3_O_4_-PAA–(HP-γ-CDs) nanobioconjugates in accordance with the analysis in Section 3.1 for use in superparamagnetic hyperthermia in vitro without toxicity and with maximum efficiency. This is presented in the next section.

#### 3.3.1. Characterization of Fe_3_O_4_-PAA Ferrimagnetic Nanoparticles

##### X-ray Diffraction of Fe_3_O_4_-PAA Nanoparticles

**Figure 5:**
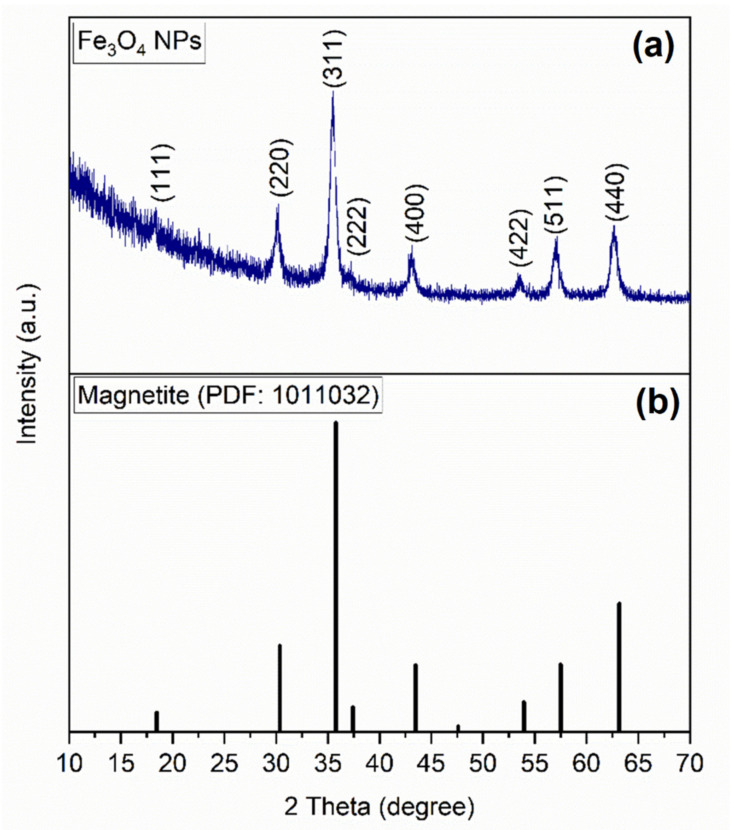
(**a**) XRD spectra of the Fe_3_O_4_-PAA nanoparticles; (**b**) XRD line spectra according to the standard sheet.

Figure 5a exhibits the XRD spectra recorded for the synthesized nanoparticles.

**Figure 6:**
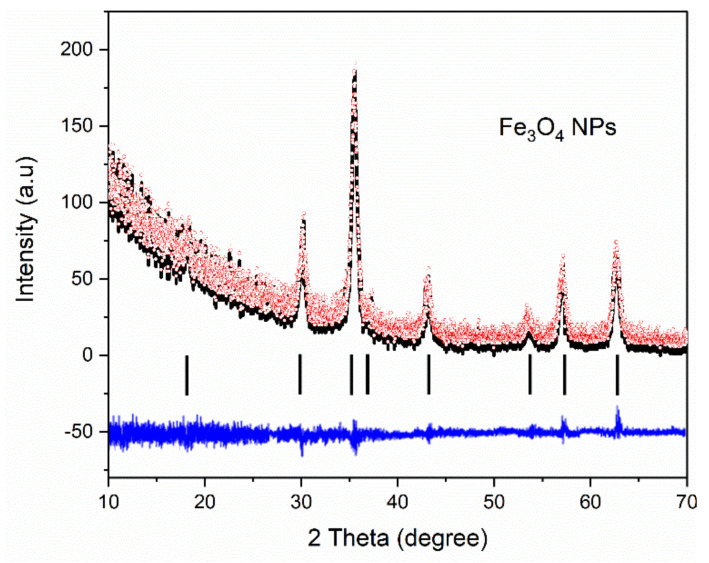
Rietveld refinement of the Fe_3_O_4_-PAA nanoparticles.

On the XRD pattern illustrated in the figure, the diffraction peaks resulting from the analysis are presented. The peaks are located at the theta angle of 18.35°, 30.08°, 35.54°, 37.42°, 43.15°, 53.37°, 57.41°, and 62.82°. For each peak, the corresponding (hkl) indices from the record corresponding to magnetite (PDF file 1011032) from the database International Centre for Diffraction Data Powder Diffraction File (ICDD PDF) 4 + 2019) were allocated. According to this correspondence between the spectra and the existing data, it can be said that the analyzed sample contains magnetite as a single phase. In the case of magnetite synthesized by co-precipitation by Kim et al. [55], on the diffraction spectra, a spinel structure with six diffraction peaks at (220), (311), (400), (422), (511), and (440) was identified. Comparing the indices and the position of the peaks with the ones obtained by us, we can conclude that the spectra show the existence of magnetite as a single phase. Magnetite was identified by XRD in other studies [59,60,61,62] and had the same diffraction peak allocation and almost identical allure. Furthermore, a Rietveld refinement was carried out (Figure 6) using the FullProf software (version July-2017, developed by J. Rodriguez-Carvajal).

Using the Debye–Sherrer equation:

(7) $$D_{XRD}=\frac{0.9\lambda }{\beta \cos\theta }$$

we calculated the mean crystallite size from the most intense diffraction peak located at 35.54°. The observables in Equation (7) are as follows: *D_XRD_* (nm) is the mean crystallites, *β* (in radians) is the full width at half of the maximum, λ (nm) is the wavelength of the X-ray, and *θ* (in radians) is the Bragg angle. From the calculation, it was found that the mean D_XRD_ is 15.9 nm.

##### Fourier Transformed Infrared Spectroscopy of Fe_3_O_4_-PAA Nanoparticles

**Figure 7:**
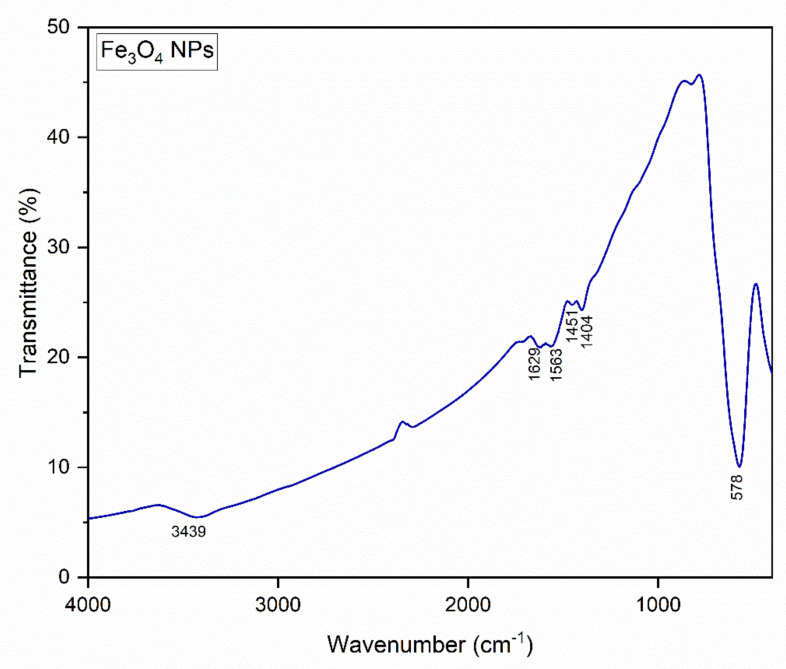
FT-IR spectra of the Fe_3_O_4_-PAA nanoparticles.

The FTIR spectra are illustrated in Figure 7. The spectrum presents a very intense absorption band located at 578 cm^−1^. Additionally, some weak bands in the 1350–1650 cm^−1^ range and a wide band at around 3400 cm^−1^ are observed. The intense band located at 578 cm^−1^ has a narrow aspect, and can be attributed to the Fe–O stretching vibration in Fe_3_O_4_. Thus, it can be said that both characterization methods, XRD and FT-IR, agree and returned the same result: the formation of Fe_3_O_4_.

Cursaru et al. [62] noted that on samples obtained using hydrothermal method, absorption bands located in the range 579–602 cm^−1^ can be attributed to Fe–O stretching vibration, thus confirming the presence of magnetite. Another study [55] presented spectra with two strong bands located at 630 and 576 cm^−1^, which were attributed to the Fe–O bond of Fe_3_O_4_. Bordbar et al. mentioned that the presence of Fe_3_O_4_ nanoparticles can be observed on FTIR spectra by a wide strong band in the 580–630 cm^−1^ range corresponding to Fe–O bond. In particular, for bulk magnetite, the absorption band is located at 576 cm^−1^ [63]. All this data from the literature supports the affirmation that we made regarding the allocation of the band located at 578 cm^−1^ to Fe–O vibration in magnetite.

The very weak bands in the domain 1350–1650 cm^−1^ can be attributed to the polyacrylic acid (PAA) that was adsorbed on the nanoparticles [64]. In addition, the band located at 3439 cm^−1^ is allocated to the –OH stretching vibration.

##### Transmission Electron Microscopy of Fe_3_O_4_-PAA Nanoparticles

**Figure 8:**
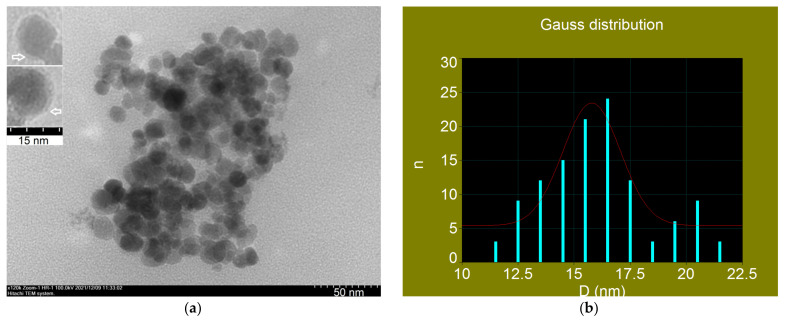
(**a**) TEM image with HR-TEM image inset (the arrow marks the PAA layer on the surface of nanoparticles), and (**b**) histogram (blue bar) and distribution function (red curve) of Fe_3_O_4_-PAA nanoparticles.

The image of the high-resolution transmission electron microscopy (HR-TEM) recorded for the Fe_3_O_4_-PAA nanoparticles is shown in Figure 8a. The result shows that nanoparticles have shapes that are close to spherical, with a distribution of nanoparticle sizes in the range of ~11–21 nm. The rather spherical shape of the nanoparticles obtained by us is an important result for our experiments of superparamagnetic hyperthermia (see Section 3.2) because elongated nanoparticles would imply an additional magnetic anisotropy (shape magnetic anisotropy) [65], depending on the degree of ellipticity of the nanoparticles. This would decrease the maximum specific loss power in magnetic hyperthermia [30] and, implicitly, the heating temperature, which will decrease the efficiency of the magnetic hyperthermia.

Due to the small thickness and low density of the polyacrylic acid layer surrounding the Fe_3_O_4_ nanoparticles, the organic shell cannot be seen in the electron microscopy image [66]. Additionally, where the nanoparticles are better dispersed to not form agglomerates, the PAA layer on the surface of the nanoparticles can be seen in the large-scale image (see the inset image in Figure 8a). The average thickness of this layer is of the order of 1 nm and is in good agreement with the value considered by us in the computational simulation.

Figure 8b shows the histogram of the diameter distribution of nanoparticles (n) in the sample. For the graphical representation, the mid-range values of the 1nm interval between two consecutive integer values of the nanoparticle diameters were taken into account, as shown in the diagram (line blue). The fitting of the experimental data leads to the Gaussian function of the distribution the diameters (D) of nanoparticles (red curve), with a most probable diameter of 15.8 nm and a standard deviation σ = 1.2 nm. The result obtained is in very good agreement with the one obtained from XRD (X-ray Diffraction of Fe_3_O_4_-PAA Nanoparticles Section), where the mean diameter is 15.9 nm.

##### Dynamic Light Scattering and Zeta Potential of Fe_3_O_4_-PAA Nanoparticles

**Figure 9:**
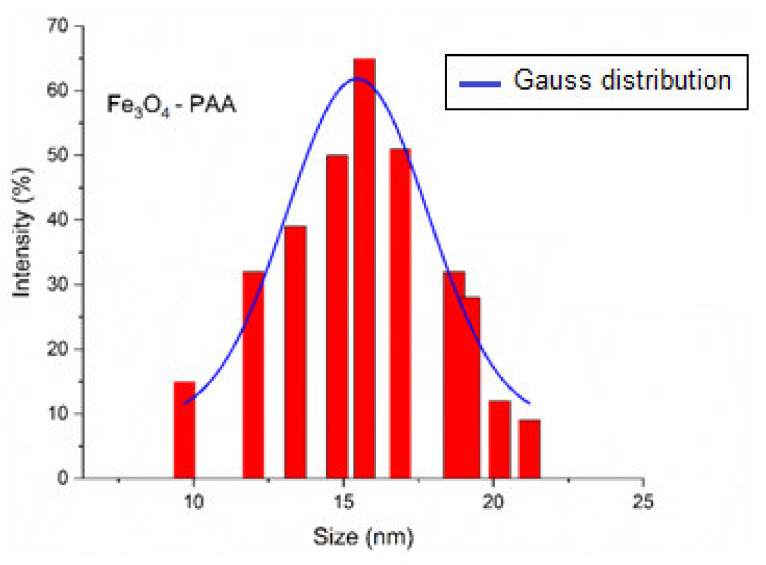
DLS for Fe_3_O_4_-PAA nanoparticles.

From the DLS determination, it was found that the mean hydrodynamic diameter of the analyzed sample is 17.5 ± 1.4 nm (Figure 9). This result is in agreement with the mean diameter of the Fe_3_O_4_ nanoparticles determined from the TEM pattern. The zeta potential determined for the sample is −17.2 mV.

##### Magnetic Behavior of Fe_3_O_4_-PAA Nanoparticles

For magnetic hyperthermia experiments, it is very important to know a priori the magnetic behavior of nanoparticles in the magnetic field, namely where it is superparamagnetic. The theoretical study of superparamagnetic hyperthermia (Section 2.2) is based on the fact that nanoparticles are superparamagnetic, as used in Section 3.2. However, this issue must also be verified experimentally.

**Figure 10:**
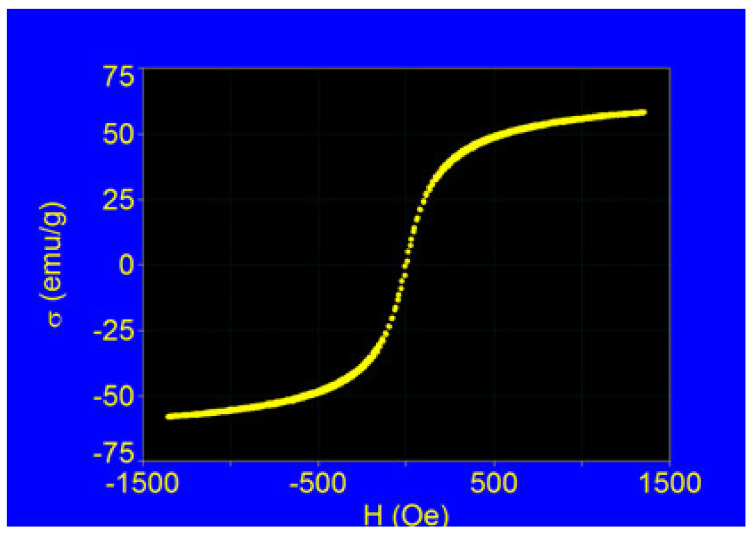
Magnetization curve of the Fe_3_O_4_-PAA nanoparticles in the external magnetic field.

In order to check this, we recorded the experimental curve of Fe_3_O_4_-PAA nanoparticles in powder form (the most unfavorable case, when the nanoparticles were not dispersed as in the case of practical magnetic hyperthermia) in the external magnetic field close to saturation. The experimental magnetization curve is shown in Figure 10.

The result obtained shows that our sample, which consisted of nanoparticles with an average diameter of ~16 nm, has no hysteresis in the external magnetic field (having a coercive field Hc~0) and the magnetic behavior is superparamagnetic [48] even for the maximum applied magnetic field of ~1400 Oe. However, in experimental magnetic hyperthermia, magnetic fields of significantly lower amplitudes than our experiment are usually used (usually below 300–400 Oe, or 25–30 kA/m (in SI units)) [23]. So, in lower magnetic fields the superparamagnetic behavior fulfils the magnetization much better depending on the amplitude of the magnetic field (σ–H) following the Langevin function [49]. This is a very important experimental result, which confirms that the Néel–Brown superparamagnetic relaxation model used in our study in Section 3.2 can be applied for our sample (Fe_3_O_4_-PAA nanoparticles).

Thus, the results obtained from the analysis in Section 3.2 can be used to optimize superparamagnetic hyperthermia in its practical implementation: in vitro and then in vivo and clinical trials in the future in order to obtain the maximum efficiency.

However, the specific saturation magnetization (σ) of Fe_3_O_4_ nanoparticles coated with PAA (Fe_3_O_4_-PAA) with a mean core diameter of ~16 nm is 58.12 emu/g. This is significantly lower than the specific saturation magnetization of bulk Fe_3_O_4_, which is 92 emu/g [46]. We attribute this decrease in the saturation magnetization of our sample both to the surface effects that exist in the case of nanoparticles [67,68,69], which lead to a decrease in the saturation magnetization, and to the presence of the organic (non-magnetic) layer of PAA on the surface of the nanoparticles, which decreases the volume packing fraction of Fe_3_O_4_ ferrimagnetic nanoparticles in the sample. However, in the case of our sample, the packing fraction remains high, with this being ε = 0.63, which is a great advantage in magnetic hyperthermia for the efficient heating of nanoparticles. Of course, in magnetic hyperthermia experiments, where nanoparticle suspensions are usually used, the packing fraction will be reduced more. However, it should be noted that in the case of our Fe_3_O_4_-PAA nanoparticles, the packing fraction of nanoparticles is quite high, which leads to an increase in the power efficiency in magnetic hyperthermia compared to other larger biostructures on the surface of nanoparticles, which considerably reduces the packing fraction, and thus decreases the efficiency in magnetic hyperthermia.

In addition, from the curve in Figure 8, the specific initial magnetic susceptibility (χ_i_), which is of interest in magnetic hyperthermia (Equations (1), (2), and (6)), was found to be 0.32 emu/Oe.g, respectively, 10.4 in SI units. In our test, this value is very good for magnetic hyperthermia because the initial magnetic susceptibilities are usually of the order of a few SI units for other ferrimagnetic nanoparticles. The high value of magnetic susceptibility in the case of our sample, according to Equation (2), is due to the large size of the nanoparticles (diameter D) (the average diameter of the nanoparticles being ~16 nm) in addition to the packing fraction (ε).

Therefore, the two parameters: the packing fraction and the initial magnetic susceptibility, are very important in our test because they allow an increase in the efficiency in superparamagnetic hyperthermia by increasing the specific loss power (SLP) and, as a result, the heating temperature of nanoparticles.

#### 3.3.2. Characterization of Fe_3_O_4_-PAA–(HP-γ-CDs) Nanobioconjugates

##### Fourier Transformed Infrared of Fe_3_O_4_-PAA–(HP-γ-CDs)

**Figure 11:**
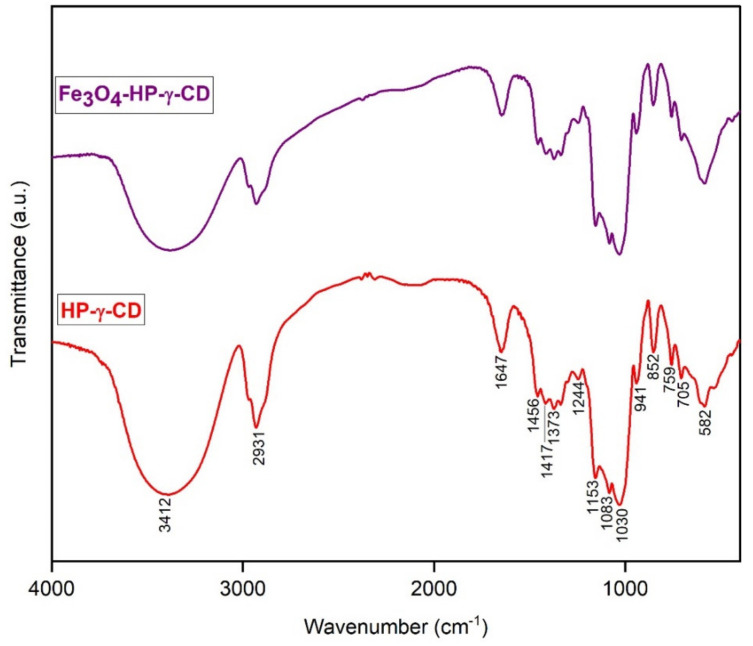
FT-IR spectra of HP-γ-CDs and Fe_3_O_4_-PAA–(HP-γ-CDs).

The nanobioconjugate of Fe_3_O_4_-PAA–(HP-γ-CDs) obtained was analyzed by means of infrared spectroscopy (FT-IR) (Figure 11). Alongside the spectra of the Fe_3_O_4_-HP-γ-CD, the spectra of the cyclodextrins are presented for comparison.

From the spectra in the figure, the similarity of the two spectra can be observed, thus indicating the formation of the nanobioconjugate. A noticeable effect is the lower intensity of the bands in the Fe_3_O_4_-HP-γ-CD spectra compared to the HP-γ-CD spectra.

Alonso et al. [70] indicated the same modification in the case of carvedilol-CD inclusion complexes; they used HP-β-CD and HP-γ-CD. Zhang et al. [71] investigated complexes from fisetin and CDs and concluded that the spectra of the inclusion complexes and of the CDs were similar. In the case of a complex formed from Fe_3_O_4_@HA@Ag and β-CD, it was found that the FT-IR spectra of the inclusion complex are very similar to the ones of the pure CDs [72]. Moreover, Atav et al. [72] suggested that in the case of the inclusion complex formation, encapsulation in the CD cavity occurs. According to Singh’s study [73], the spectra of HP-γ-CD have the same appearance as those obtained by us. Furthermore, he allocated the important bands between 3600 and 3100 cm^−1^ to OH stretching vibration and those at 2928 cm^−1^ to asymmetric CH stretching of CH_2_.

The other allocations of bands are as follows: around 1640 cm^−1^ is the deformation band of water H-O-H; the band at 1459 cm^−1^ shows the in-plane bending of CH_3_; the band at 1362 cm^−1^ corresponds to in-plane bending of CH, enolic COH, and skeletal CCC. The characteristic wide band with peaks at 1155, 1082, and 1030 cm^−1^ can be allocated to CH overtone stretching + C–O–C stretching, C–O,C–C stretching + in-plane bending of OCH, and C–O, C–C, CCO, and C–O–C stretching of glucose units, respectively. There are still few bands under 1000 cm^−1^: at 944 cm^−1^, correlated with O stretching and in-plane bending of CCH; at 855 cm^−1^, corresponding to CH out-of-plane bending of aromatic CCH and skeletal CCH; and at 753 cm^−1^, allocated to CH out-of-plane bending and skeletal C-C stretching. The position of the absorption bands presented by Singh is very similar to the ones obtained by us and are presented in Figure 11.

##### Dynamic Light Scattering and Zeta Potential Measurements of Fe_3_O_4_-PAA-(HP-γ-CDs)

**Figure 12:**
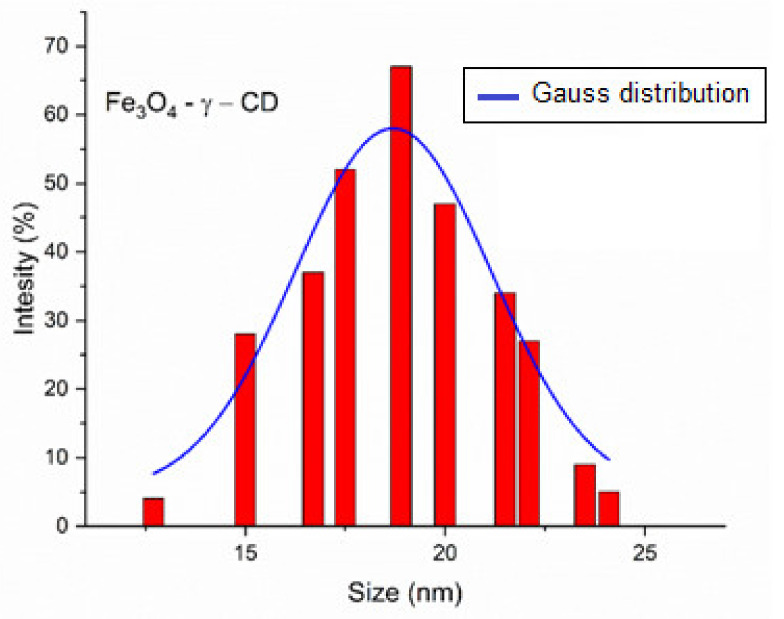
DLS for Fe_3_O_4_-PAA–(HP-γ-CDs) nanobioconjugates.

Similar to the DLS measurement for the magnetite, measurement of the nanobioconjugates was carried out. In the case of Fe_3_O_4_-HP-γ-CD, the mean hydrodynamic diameter was ~20 ± 1.7 nm (Figure 12). The difference in the mean diameter between Fe_3_O_4_ NPs and Fe_3_O_4_-PAA-(HP-γ-CDs) appears to be due to the coating layer that the cyclodextrins with PAA forms around the nanoparticles. The zeta potential measured for the nanobioconjugate was −28.6 mV.

### 3.4. Cell Viability Assessment under Standard Conditions for the Prepared Fe_3_O_4_-PAA Nanoparticles and Fe_3_O_4_-PAA–(HP-γ-CDs) Nanobioconjugates

Before using the obtained nanoparticles and nanobioconjugates to induce magnetic hyperthermia, it is necessary to evaluate their biological profile (cell viability/cytotoxicity). The cytotoxicity must comply with accepted biological limits of international standards (ISO standard). Nanoparticles that reveal toxicity exceeding the biological limit cannot be further used for magnetic hyperthermia.

In this regard, to obtain the preliminary biological profile of Fe_3_O_4_-PAA nanoparticles and Fe_3_O_4_-PAA–(HP-γ-CDs) nanobioconjugates, different concentrations (200, 500, and 1000 µg/mL) of the aforementioned test compounds were applied on human immortalized keratinocytes (HaCaT) 2D cell cultures under standard conditions ST (37 °C) for an interval of 24 and 48 h. HaCaT cells were employed because they are considered as one of the most used in vitro models. Moreover, this study is considered as a starting point for future research regarding the administration of magnetic compounds in skin cancer, including melanoma. Thus, through this study, the results obtained on healthy human keratinocytes (HaCaT cell line, i.e., the healthy skin cell line) constitute important data that will be used as a starting point in future studies.

**Figure 13:**
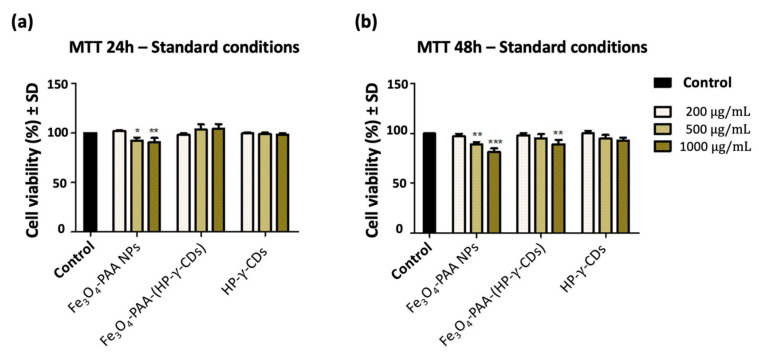
Cell viability percentage of immortalized human keratinocytes (HaCaT) exposed to test samples at different concentrations (200, 500, and 1000 µg/mL): (**a**) Cell viability at 24 h post-stimulation; (**b**) cell viability at 48 h post-stimulation. The results are expressed as the mean values of the cell viability percentage (%) normalized to control cells (cells treated with culture media) ± standard deviation (SD). One-way ANOVA analysis was applied to determine the statistical differences between test-treated cells and control, followed by Dunnett’s post-test (* *p* < 0.05; ** *p* < 0.01; *** *p* < 0.001 versus control).

The results were quantified by applying the MTT method and the cell viability percentages are presented in Figure 13.

As a general observation related to the cell viability percentage (Figure 13), it should be pointed out that the cell viability percentage of HaCaT cells after exposure to the nanobioconjugate sample Fe_3_O_4_-PAA–(HP-γ-CDs) is higher when compared to the biological effect observed after treating the HaCaT monolayers with the magnetic compound (Fe_3_O_4_-PAA NPs) uncoated with HP-γ-CD. Thus, the viability of Fe_3_O_4_-PAA–(HP-γ-CDs)-treated cells did not decrease below 98% after 24 h while the HaCaT population exposed to Fe_3_O_4_ NPs for 24 h showed decreased viability of 90.7% after exposure to the highest concentration of 1000 µg/mL Fe_3_O_4_ NPs (Figure 13a).

HP-γ-CD did not induce a cytotoxic effect because the viability of HaCaT cells was above 98% even after exposure to the concentration of 1000 µg/mL.

Incubation of the HaCaT monolayer with the test samples for a period of 48 h induced a slight decrease in the cell viability compared to the incubation interval of 24 h; however, the general pattern was maintained: the HaCaT culture presented a higher viability of 89.2% for the high concentration of 1000 µg/mL after exposure to Fe_3_O_4_-PAA–(HP-γ-CDs) while the viability recorded for the cells treated with the magnetic compound (Fe_3_O_4_-PAA NPs) uncoated with HP-γ-CD presented a slightly reduced viability of 81.4% (Figure 13b).

According to the ISO standard for the biological evaluation of medical devices (ISO Standard 10993-5: 2009) [74], a compound is considered to be cytotoxic if the cell viability is under 70%. Therefore, none of the test samples could be considered cytotoxic under the present conditions employed: a time interval of 24 and 48 h and concentrations up to 1000 µg/mL.

### 3.5. In Vitro Superparamagnetic Hyperthermia Using Biocompatible Fe_3_O_4_-PAA–(HP-γ-CDs) Nanobioconjugates

#### 3.5.1. Experimental Superparamagnetic Hyperthermia with Fe_3_O_4_-PAA Nanoparticles: T–t Temperature Diagram in Calorimetric Conditions

**Figure 14:**
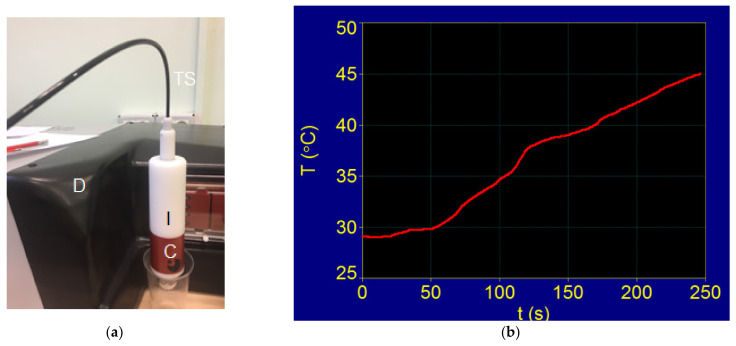
(**a**) Calorimetric system for measuring the temperature in a magnetic suspension with nanoparticles during magnetic hyperthermia; D: magnetic hyperthermia driver, C: inductor coil, TS: fiber optic temperature sensor, I: thermal insulation surrounding the vacuum chamber; (**b**) T–t experimental diagram of the magnetic suspension of Fe_3_O_4_-PAA for 10 mg/mL.

Once we determined that the Fe_3_O_4_-PAA nanoparticles obtained by us did not produce cellular toxicity, the next step was to investigate whether the increase in the temperature of the suspension with magnetic nanoparticles, after applying the alternative magnetic field with appropriate parameters for H and f, reached the value required in magnetic hyperthermia for the destruction of tumors (~42.5 °C), and the duration of this process. For this, the Fe_3_O_4_-PAA nanoparticles were dispersed in water (close to saline) at a ratio of 10 mg/mL to achieve superparamagnetic hyperthermia, considering that such a concentration is used in practical magnetic hyperthermia [14,75]. Calorimetric measurements (see the other details in Section 2.5) of the increase temperature in the suspension with nanoparticles (Figure 14a) for the recording over time led to the result shown in Figure 14b.

The graph shows the increase in the temperature (T) of the nanoparticle suspension during (t) which superparamagnetic hyperthermia occurred (T–t diagram). A magnetic field with an amplitude of 15.92 kA/m and frequency of 312.2 kHz produced by the F3 driver for magnetic hyperthermia was used to obtain superparamagnetic hyperthermia. These values ensure that the condition [76]:

(8) $$H\times f<5\times 10^{9}\;\;\left(Am^{-1}Hz\right)\;$$

is met in order not to exceed the permissible biological limit for the magnetic field, which would affect healthy tissues. The temperature was measured with a precision temperature sensor with fiber optics (Figure 14a) under adiabatic conditions (the glass tube in which the sample was inserted was separated from the external environment by means of a vacuum chamber and very good thermal insulation; so, the heat transfer from the sample to the outside environment can be neglected).

The result obtained shows a very important aspect for magnetic hyperthermia, namely, that the temperature that would be required in magnetic hyperthermia of approximately 42.5 °C is reached very quickly in just a few minutes (approximately 3.3 min). According to this experimental result, it is confirmed that our sample is very suitable for use in superparamagnetic hyperthermia in terms of the specific loss power and heating temperature.

#### 3.5.2. Superparamagnetic Hyperthermia with Fe_3_O_4_-PAA–(HP-γ-CDs) Nanobioconjugates in Cells Culture: T–t Temperature Diagram of In Vitro Conditions

**Figure 15:**
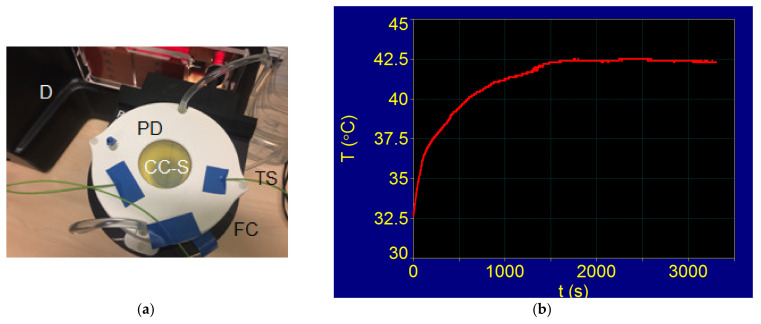
(**a**) Coil magnetic hyperthermia generator for in vitro studies; CC-S: cell culture with Fe_3_O_4_-PAA–(HP-γ-CDs) nanobioconjugates; (**b**) T–t diagram for cell culture with Fe_3_O_4_-PAA–(HP-γ-CDs) nanobioconjugates.

Testing of the obtained Fe_3_O_4_-PAA–(HP-γ-CDs) nanobioconjugates to achieve superparamagnetic hyperthermia in vitro was performed on a human immortalized keratinocytes cell line (HaCaT). For this, the F3 magnetic hyperthermia driver (D) with a flat induction coil for in vitro conditions (FC) was used (Figure 15a). The standard conditions required for cell culture (CC) were used for this study. The magnetic field in this case was applied to the cell culture with Fe_3_O_4_-PAA–(HP-γ-CDs) nanobioconjugates (CC-S) via flat induction coils FC, which created a uniform magnetic field in the sample CC-S, with a value of 15.92 kA/m and a frequency of 321.2 kHz, as in calorimetric conditions. Cell culture with magnetic nanoparticles in a standard crucible with an approximately 3 cm diameter was introduced centrally in a petridish (PD). This system allows the maintenance of standard conditions for cell culture with nanobioconjugates during the experiment (sterile culture medium, controlled atmosphere, and temperature of 37 °C). The temperature of the cellular environment was measured with a temperature sensor equipped with fiber optics (TS), with an acquisition step of 0.1 °C.

The registration of experimental data and the control over the experiment were carried out automatically, with specialized professional software and a data acquisition system. The temperature in the magnetic hyperthermia experiment was controlled by the amplitude of the applied magnetic field, so that the temperature remained constant at the value provided in the experiment of 42.5 °C, which is required in magnetic hyperthermia.

For the experimental testing of magnetic hyperthermia with Fe_3_O_4_-PAA–(HP-γ-CDs) nanobioconjugates on cell cultures, an exposure time of 30 min was used, starting from the moment when the temperature in the experiment reached the value corresponding to the magnetic hyperthermia of 42.5 °C. The concentration used in the experiment was 10 mg/mL Fe_3_O_4_-PAA–(HP-γ-CDs) nanobioconjugates in suspension, corresponding to an equivalent cell volume.

The experimentally recorded T–t diagram in this case is shown in Figure 15b. The figure shows that after applying the magnetic field at the initial moment t = 0, the temperature required in magnetic hyperthermia (42.5 °C) is reached after approximately 23 min; there is a rapid increase in the first minutes and then a slowing down, with a tendency towards saturation at the set value of 42.5 °C.

The duration and shape of the variation of the temperature curve until the value of 42.5 °C is reached are mainly due to the automatic electronic system of the generator, which, through a feedback reaction, regulates the controlled increase in the temperature at the experiment set value of 42.5 °C by gradually decreasing the amplitude of the magnetic field. When this temperature of 42.5 °C is reached, it is kept constant in the experiment by small variations in +/− ΔH of the magnetic field around an average value H_0_. Another cause that contributes to the delay in reaching the temperature threshold of 42.5 °C is also that in this case, the sample is no longer well isolated (by vacuum) from the external environment (Figure 15a), as in the previous calorimetric experiment (Figure 14a), with small heat dissipation to the outside environment occurring. Additionally, the cellular environment in which the nanobioconjugates of Fe_3_O_4_-PAA–(HP-γ-CDs) were dispersed, with different thermal conductivities, has an influence on how the temperature rises to reach the temperature of 42.5 °C.

However, the above factors do not influence the results of in vitro magnetic hyperthermia as long as the temperature of 42.5 °C is reached, even in a short time interval (approximately 23 min), and a constant temperature can be maintained as needed in the in vitro superparamagnetic hyperthermia experiment.

Thus, in this superparamagnetic hyperthermia experiment, the temperature was kept constant at 42.5 °C (±0.2 °C) for approximately 30 min to monitor the effect of the temperature generated by Fe_3_O_4_-PAA–(HP-γ-CDs) nanobioconjugates on healthy HaCaT cells. The effect of in vitro superparamagnetic hyperthermia with Fe_3_O_4_-PAA–(HP-γ-CDs) nanobioconjugated on HaCaT cells is presented in the next section.

#### 3.5.3. Cell Viability Assessment of HaCaT Cells under Magnetically Induced Hyperthermia Conditions

**Figure 16:**
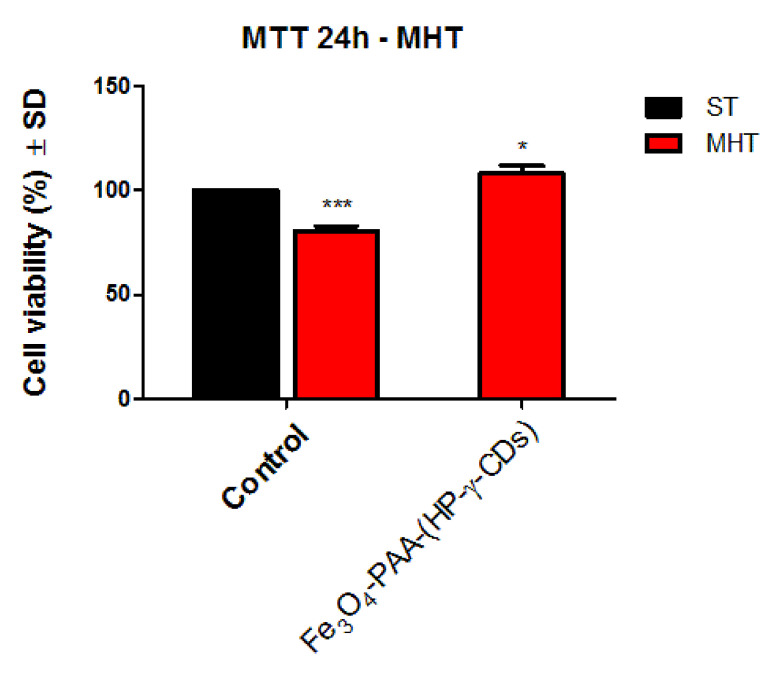
Cell viability percentage of HaCaT cells maintained under standard (ST) and magnetic hyperthermia (MHT) parameters or treated with 10,000 µg/mL of Fe_3_O_4_-PAA–(HP-γ-CDs) under MHT conditions. Data are presented as the percentage of the viable cell population under MHT (stimulated or unstimulated with test sample) normalized to the cell viability rate of the control cells (cells treated with culture medium and maintained under standard conditions at 37 °C) ± standard deviation (SD). One-way ANOVA analysis was applied to determine the statistical differences between MHT-treated cells and control cells, followed by Dunnett’s post-test (* *p* < 0.05; *** *p* < 0.001 versus control).

Based on the good cell viability results obtained for HP-γ-CDs and Fe_3_O_4_-PAA–(HP-γ-CDs) nanobioconjugates (Section 3.4), to expend the applicability of these samples to superparamagnetic hyperthermia, the cell viability of the HaCaT cells was determined after inducing approximately 30 min of hyperthermic conditions (by applying an alternative magnetic field). In vitro hyperthermia was obtained after increasing the concentration of nanobioconjugates 10 times, respectively, from 1000 to 10,000 μg/mL. The in vitro results obtained under magnetically induced hyperthermia (MHT) are shown in Figure 16.

The results revealed that the viability of control MHT–HaCaT cells treated only with cellular medium and exposed to the alternating magnetic field (AMF) did not decrease below 80%; thus, it could be stated that the magnetic parameters employed to induce the hyperthermic conditions are not cytotoxic for the HaCaT population.

However, the morphology of the HaCaT control cells was not affected after exposure to the magnetic field and induction of magnetic hyperthermia conditions, both immediately after exposure and 24 h after. As presented in Figure S1 (Supplementary Materials), the cells present the same morphological characteristics: acicular appearance, high confluence, and multiple intercellular connections. Induction of magnetic hyperthermia in HaCaT cells treated with a concentration of 10,000 µg/mL Fe_3_O_4_-PAA–(HP-γ-CDs) under hyperthermic conditions induced a slight stimulatory effect on the cell viability; this might be explained by the interference of the MTT reagent with the high concentration of Fe remaining attached to the cell culture, even after washing the cell monolayer with PBS three times, as recommended in the literature [5]. However, no significant morphological alterations were recorded, as presented in Figure S1.

Nevertheless, for successful in vitro magnetic hyperthermia (MHT) that can provide reliable results and is suitable for extrapolation to the in vivo scenario, several aspects must be considered, as the results reported in the literature vary widely. Regarding this aspect, Vilas-Boas et al. [77] discussed the influence of cell culture set-up on the expected outcomes, such as: adherent cell cultures are usually more sensitive to MHT, compared to cell suspension, while cell clusters with a volume of at least 1 mm^3^ seem to provide the most suitable microenvironment to better understand the cytotoxic potential of MHT. In other words, complex three-dimensional (3D) spheroid structures obtained from various human cell lines may provide closer results to those obtained on animal models with xenographs of tumorigenic human cell lines.

Thus, it can be stated that superparamagnetic hyperthermia with Fe_3_O_4_-PAA–(HP-γ-CDs) nanobioconjugates can be achieved in vitro at 42.5 °C without inducing toxicity of healthy cells at a concentration of up to 10,000 μg/mL nanoconjugates in suspension. This result is very important for future alternative cancer therapy.

Given the results reported in this paper, our future studies will focus on the application of superparamagnetic hyperthermia using Fe_3_O_4_-PAA–(HP-γ-CDs) nanobioconjugates in vitro and then in vivo cancer therapy for cancers that have a high incidence in the population, such as breast and skin cancer, including melanoma.

## 4. Conclusions

Based on our theoretical predictions of molecular docking, to find the optimal nanobiostructure for bioconjugation of Fe_3_O_4_ ferrimagnetic nanoparticles with cyclodextrins, and computational simulation of the maximum specific loss power in superparamagnetic hyperthermia, we designed and obtained the appropriate magnetic nanobiostructure for use without cellular toxicity and with increased efficiency in superparamagnetic hyperthermia. Thus, we obtained core-shell Fe_3_O_4_-PAA–(HP-γ-CDs) nanobioconjugates with a magnetic core mean diameter of ~16 nm for Fe_3_O_4_ nanoparticles coated with an organic layer of PAA–(HP-γ-CDs), which ensured very good cell biocompatibility on a human HaCaT cell line (cell viability of around 100% for concentrations up to 1000 μg/mL). In addition, the organic shell at the surface of the Fe_3_O_4_ ferrimagnetic core was quite thin, only ~2 nm. This will increase the efficiency of obtaining the hyperthermic effect in magnetic hyperthermia compared to other large biostructures (e.g., liposomes), where the efficiency is low.

Our results reported in this paper demonstrate that Fe_3_O_4_ nanoparticles with an average diameter of ~16 nm bioconjugated with branched γ-CDs (Fe_3_O_4_-PAA–(HP-γ-CDs)) have no cellular toxicity up to a concentration of 10,000 μg/mL in suspension and can be used successfully in high-efficiency superparamagnetic hyperthermia. Under the in vitro experimental conditions used by us, the required temperature of 42.5 °C in the magnetic hyperthermia of tumors was reached in a relatively short time of only 23 min to not affect healthy tissues in therapy. In vitro application of superparamagnetic hyperthermia for 30 min using Fe_3_O_4_-PAA–(HP-γ-CDs) nanobioconjugates did not affect human immortalized keratinocytes (HaCaT) 2D cells.

Additionally, our study established the conditions under which superparamagnetic hyperthermia can be optimally applied in vitro using Fe_3_O_4_-PAA–(HP-γ-CDs) nanobioconjugates, both more effectively and without cellular toxicity, in order to be used in future cancer alternative therapy.

## Figures and Tables

**Table 1:** The parameters of the alternating magnetic field and characteristic observables of Fe_3_O_4_-PAA–(HP-γ-CDs) nanoparticles; M_s_ is the spontaneous magnetization of Fe_3_O_4_, K is the magnetocrystalline anisotropy constant of Fe_3_O_4_, ρ is the density of the nanoparticle material, d is the (mean) thickness of the organic shell (PAA–(HP-γ-CDs) on the surface of core Fe_3_O_4_ nanoparticles, and η is the viscosity coefficient of the dispersion medium (saline).

Observables	H (kA/m)	f (kHz)	M_s_ (kA/m)	K (kJ/m^3^)	ρ (×10^3^ kg/m^3^)	2d (nm)	η (kg/ms)	ε	D (nm)
Values	16	200–400	480	11	5.24	3.6	7 × 10^−4^	0.1	5–25

**Table 2:** Free binding energy values of PA20n (PAA) following docking into various cyclodextrins.

Ligand	CD	∆G (kcal/mol)
PA20n	β-CD	−4.0
	γ-CD	−4.4
	HPBCD	−4.9
	HPGCD	−5.0
	Mono-OH-SGM	−4.9

**Table 3:** The values of the maximum specific loss power (SLP)_M_ and the nanoparticle diameter (D_o_) corresponding to (SLP)_M_ for three frequencies in the case of fixed nanoparticles.

H (kA/m)	f (kHz)	(SLP)_M_ (W/g)	D_o_ (nm)
16	200	47.95	16.9
16	321	75.77	16.5
16	400	93.69	16.3

**Table 4:** The values of the maximum specific loss power (SLP)_M_ and the nanoparticle diameter (D_o_) corresponding to (SLP)_M_ for three frequencies in the case of mobile nanoparticles.

H (kA/m)	f (kHz)	(SLP)_M_ (W/g)	D_o_ (nm)
16	200	48.44	17.1
16	321	76.31	16.6
16	400	94.18	16.4

## Data Availability

Not applicable.

## Supplementary Materials

The following supporting information can be downloaded at: https://www.mdpi.com/article/10.3390/nano12152577/s1, Figure S1: Morphological aspects of immortalized human keratinocytes (HaCaT), before and after inducing magnetic hyperthermia (MHT). The photos were taken using an inverted microscope (Optika Microscopes Optikam Pro Cool 5 and Optika View), employing magnification of 10× magnification; nd - not determined.
