# Supplementary material for: Fe3O4-PAA–(HP-γ-CDs) Biocompatible Ferrimagnetic Nanoparticles for Increasing the Efficacy in Superparamagnetic Hyperthermia
Source: Nanomaterials (Basel). 2022 Jul 27;12(15):2577. doi: 10.3390/nano12152577 (PMC9370715; doi:10.3390/nano12152577)
Supplement: Supplementary file 1 [file nanomaterials-12-02577-s001.zip › nanomaterials-1756712-supplementary.pdf]

Supplementary Materials

Fe_3O_4-PAA–(HP-γ-CDs) Biocompatible Ferrimagnetic Nanoparticles for Increasing the Efficacy in Superparamagnetic Hyperthermia

Costica Caizer [1], Isabela Simona Caizer [1,2,3], Roxana Racoviceanu [4,5,*], Claudia Geanina Watz [5,6,*], Marius Mioc [4], Cristina Adriana Dehelean [4,5], Tiberiu Bratu [2] and Codruța Soica [4,5]

1 Department of Physics, Faculty of Physics, West University of Timisoara, 300223 Timisoara, Romania; costica.caizer@e-uvt.ro (C.C.); isabela.caizer@umft.ro (I.S.C.)
2 Department of Plastic and Reconstructive Surgery, Faculty of Medicine, "Victor Babes" University of Medicine and Pharmacy of Timisoara, 300041 Timisoara, Romania; tiberiu.bratu@umft.ro
3 Department of Clinical Practical Skills, Faculty of Medicine, "Victor Babes" University of Medicine and Pharmacy of Timisoara, 300041 Timisoara, Romania
4 Department of Pharmaceutical Chemistry, Faculty of Pharmacy, "Victor Babes" University of Medicine and Pharmacy of Timisoara, 300041 Timisoara; Romania marius.mioc@umft.ro (M.M.); cadehelean@umft.ro (C.A.D.); codrutasoica@umft.ro (C.S.)
5 Research Centre for Pharmaco-Toxicological Evaluation, "Victor Babes" University of Medicine and Pharmacy of Timisoara, 300041 Timisoara, Romania
6 Department of Pharmaceutical Physics, Faculty of Pharmacy, "Victor Babes" University of Medicine and Pharmacy of Timisoara, 300041 Timisoara, Romania
* Correspondence: babuta.roxana@umft.ro (R.R.); farcas.claudia@umft.ro (C.G.W.)

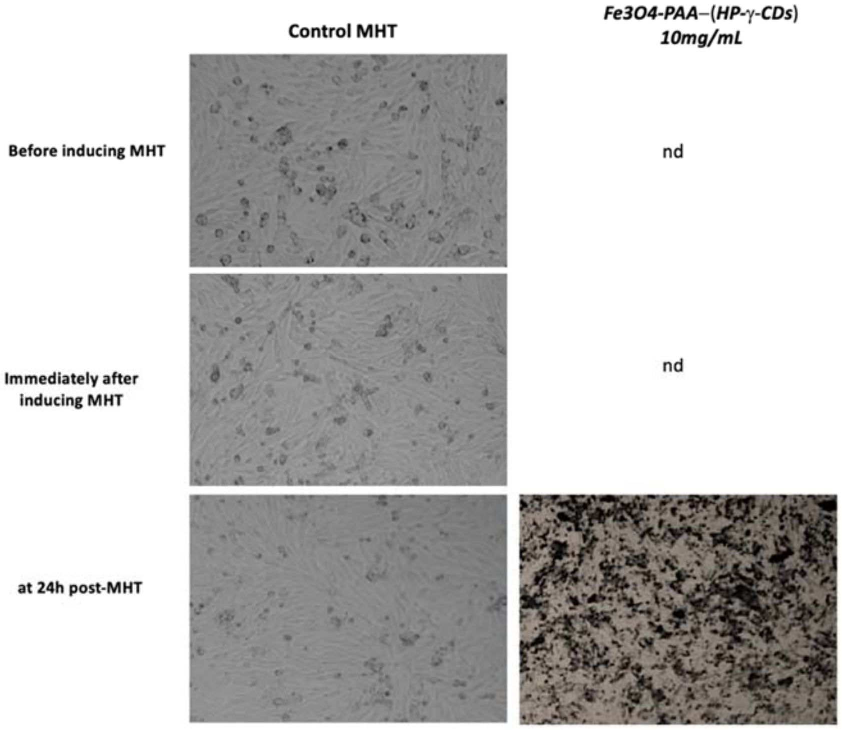

Figure S1. Morphological aspects of immortalized human keratinocytes (HaCaT), before and after inducing magnetic hyperthermia (MHT). The photos were taken using an inverted microscope (Optika Microscopes Optikam Pro Cool 5 and Optika View), employing magnification of 10x magnification; nd - not determined.

The morphology of the cells treated with 10 mg/mL of Fe_3O_4-PAA-(HP-ϒ-CDs) under hyperthermic conditions could be observed after 24h post-exposure, when multiple washes steps of the cell culture plate with 1x PBS (phosphate buffer saline) were employed and a significant part of the compound was removed, thus allowing the capturing of images with an inverted microscope. Before performing the washing steps, the cell morphology could not be determined (nd), due to the high concentration of Fe_3O_4-PAA-(HP-ϒ-CDs) that induced high opacity.

However, at 24h post-MHT, the images indicate that no significant cell morphology alterations occurred when comparing the control cells with the cells treated with Fe_3O_4-PAA-(HP-ϒ-CDs); a high cell confluence being observed for both groups: control cells and Fe_3O_4-PAA-(HP-ϒ-CDs)-treated cells, maintained under the same MHT parameters.
